# Phase I dose escalation study of vinorelbine and topotecan combination chemotherapy in patients with recurrent lung cancer

**DOI:** 10.1186/1471-2407-7-231

**Published:** 2007-12-20

**Authors:** Matthew A Beldner, Carol A Sherman, Mark R Green, Elizabeth Garrett-Mayer, Uzair Chaudhary, Mario L Meyer, Andrew S Kraft, Alberto J Montero

**Affiliations:** 1 Division of Medical Oncology/Hematology, Hollings Cancer Center, Medical University of South Carolina, 96 Jonathan Lucas Street, Charleston, SC 29425, USA; 2 Biostatistics, Bioinformatics & Epidemiology, Hollings Cancer Center, Medical University of South Carolina, 86 Jonathan Lucas Street, Charleston, SC 29425, USA

## Abstract

**Background:**

A platinum doublet is the current standard treatment for good performance status patients with advanced non-small cell lung cancer (NSCLC) and extensive stage small cell lung cancer (SCLC) with good performance status. However, platinum-based treatment may be associated with significant toxicities, therefore alternative platinum-free combinations should be investigated. Topotecan is a topoisomerase I inhibitor that exerts its cytotoxic effect through stabilization of the topoisomerase I-DNA complex. Preclinical data suggests synergy between topoisomerase I inhibitors and mitotic spindle poisons. Considerable hematologic toxicities have been reported with topotecan dosed for 5 consecutive days in combination with vinorelbine. Therefore, the aim of this study was to evaluate the optimal dosage and the maximal tolerated dose (MTD) of topotecan and vinorelbine in patients with relapsed  or refractory non-small cell or small cell lung cancer administered on  an alternate dosing schedule.

**Methods:**

From February, 2004 to March, 2007 eighteen patients with advanced or recurrent NSCLC or SCLC previously treated with chemotherapy were enrolled. Patients were heavily pretreated with 22% having received at least 3 prior lines of chemotherapy. Vinorelbine was administered at a fixed dose (20 mg/m^2^) and topotecan at escalating doses (2, 2.5, 3, 3.5, and 4 mg/m^2^) on days 1 and 8 every 21 days.

**Results:**

The MTD was not reached in any of the 5 cohorts, with only one dose limiting toxicity (DLT) occurring in cohort 4. Non-hematological toxicities were manageable. One patient had a partial response with four patients (27%) achieving stable disease. The median progression-free and overall survival for all patients, were 2.7 months (95% CI: 1.6, 9.1) and 10.5 months (95% CI: 4.2, 22.7), respectively.

**Conclusion:**

Vinorelbine and topotecan administered on days 1 and 8 every 21 days is well tolerated without any DLT seen with previously investigated topotecan schedules. This doublet provides a potentially active non-platinum containing doublet for the treatment of patients with advanced SCLC and NSCLC. Vinorelbine and topotecan should therefore be investigated in subsequent phase II studies at a dose of 20 mg/m^2 ^and 4 mg/m^2^, respectively.

**Trial Registration Number:**

NCT00287963.

## Background

Advanced lung cancer remains a clinical challenge, with median survival estimated in months, and one-year survival of less than 10% in patients treated with best supportive care alone[1,2]. In patients with advanced non-small cell lung cancer (NSCLC) single agent therapy with cisplatin results in a modest clinical benefit and its use is an independent prognostic variable predicting superior survival[1,3]. The addition of a third generation cytotoxic agent, including either vinorelbine, gemcitabine, docetaxel or paclitaxel, to cisplatin results in improved response rates and almost a doubling of 1-year survival [3,4]. Multiple regimens have been compared, using a platinum, either carboplatin or cisplatin, as one agent in combination with another cytotoxic agent. In the ECOG 1594 trial, where patients with stage IV NSCLC were treated with four different platinum containing doublets, no significant differences in overall response rates or overall survival between them was observed [4]. Likewise, in small cell lung cancer (SCLC) combination platinum chemotherapy regimens are commonly utilized in part due to their high response rates as first line therapy, particularly in patients with limited stage disease [5]. However, platinum-based treatment is associated with significant toxicities, therefore development of alternative platinum free chemotherapy combinations are warranted.

Topotecan is a topoisomerase I inhibitor that exerts its cytotoxic effect through stabilization of the topoisomerase I-DNA complex, thereby causing single-stranded DNA breaks during replication [6]. Topotecan administered by intravenous infusion daily for 5 days of a 21-day cycle at a dose of 1.5 mg/m^2 ^is an established treatment in recurrent small-cell lung cancer (SCLC) [7,8]. In second-line, extensive stage SCLC, response rates (ORR) up to 22% have been reported [5,9-11]. The standard 5-day topotecan regimen approved for use in recurrent SCLC is also an established treatment for relapsed ovarian cancer [12].

One of the drawbacks of the standard topotecan schedule (1.5 mg/m^2^/day via a 30-minute i.v. injection on days 1–5 of a 21-day cycle) is that it is associated with significant myelosuppression with approximately 70% and 29% of patients experiencing grade 4 neutropenia and thrombocytopenia, respectively [2]. Myelosuppression is transient, non-cumulative, and manageable, although approximately 5% of patients develop febrile neutropenia. The major non-hematologic toxicities include fatigue, constipation, and nausea/vomiting [13,14]. Weekly administration of topotecan has a different side effect profile with less neutropenia, and also permits greater dose intensity and thus greater ease of combining topotecan with other anti-neoplastic agents [15,16].

Vinorelbine is a semi-synthetic vinca alkaloid; its most common dose-limiting toxicity is neutropenia [17-19]. Vinorelbine is widely used in NSCLC where it has shown considerable activity either as a single agent or in combination [19,20]. Vinorelbine has been studied in both previously treated and untreated patients with SCLC [18]. The rationale for combining topotecan and vinorelbine for recurrent lung cancer is primarily based on single agent activity and different toxicity profiles. Moreover, preclinical data also suggests that topoisomerase I inhibitors and mitotic spindle poisons are a synergistic combination [21]. This suggests that the combination of these agents, both of which have independent activity in lung cancer, would be of potential utility in the salvage setting.

Previous studies in patients with untreated SCLC or NSCLC have evaluated weekly topotecan in combination with other chemotherapeutic agents including: paclitaxel; docetaxel; gemcitabine; temozolomide, oxaliplatin, cisplatin, and 5-FU/leucovorin [22-27]. Overall these studies have demonstrated that weekly topotecan in combination with various other cytotoxic agents could be safely administered to patients at doses between 1.5–4.5 mg/m^2^.

To date, two previously published phase I studies which investigated escalating doses of daily topotecan in combination with vinorelbine, indicated that topotecan could not be administered without filgrastim for 3 or 5 consecutive days at doses greater than 1.5 mg/m^2 ^or 0.85 mg/m^2^, respectively [28,29]. Therefore, a weekly dosing schedule of both vinorelbine and topotecan warrants evaluation. To our knowledge, no previous phase I study of escalating doses of weekly topotecan, on days 1 and 8, in combination with a fixed dose of vinorelbine has been previously published in patients with recurrent lung cancer (NSCLC or SCLC). The purpose of this study therefore was to determine the maximum tolerated dose (MTD) of this chemotherapy doublet in patients with recurrent lung cancer.

## Methods

### Patients

The Protocol Review Committee of the Hollings Cancer Center and the Medical University of South Carolina Institutional Review Board approved this study. A written informed consent was obtained from all subjects. Patients were enrolled from February, 2004 to March, 2007 at the Medical University of South Carolina. Patients were recruited based upon the following eligibility criteria: pathologically proven lung cancer, all histologic types of lung cancer were eligible (both small cell and non-small cell lung cancer); recurrent or progressive disease after at least one prior chemotherapy +/- radiotherapy regimen; at least 2 weeks after completion of prior radiotherapy; no prior therapy with topotecan or vinorelbine; age ≥ 18 years; ECOG performance status ≤ 2; adequate organ and marrow function defined as: absolute neutrophil count ≥ 1,500/mm3, platelets ≥ 100,000/mm^3^, total bilirubin ≤ 1.5 mg/dl, creatinine ≤ 1.5 mg/dl; non-pregnant and non-lactating; no active concurrent invasive malignancy; ability to understand and the willingness to sign a written informed consent document. Patients were excluded if they: had chemotherapy within 4 weeks prior to entering study; were currently receiving other investigational agents; had a history of allergic reactions attributed to compounds of similar chemical or biologic composition to topotecan or vinorelbine; had uncontrolled intercurrent illness including active infection, symptomatic congestive heart failure, unstable angina pectoris, cardiac arrhythmia, or psychiatric illness.

### Treatment and Dose Escalation

This was an open-label, single center, non-randomized, dose escalating phase I study. Treatment was administered on an outpatient basis. Topotecan and vinorelbine were each administered on days 1 and 8 every 3 weeks [1 cycle = 21 days] starting at doses of 20 mg/m^2^, and 2 mg/m^2^, respectively. Doses of topotecan were increased, according to a modified Fibonacci schedule, to 2.5, 3.0, 3.5, and 4.0 mg/m^2 ^(Table 1). Vinorelbine was available in 1-ml or 5-ml vials (10 mg/ml) and was diluted in any of the following solutions: 5% dextrose, 0.9% sodium chloride, 0.45% sodium chloride, 5% dextrose and 0.45% sodium chloride, Ringer's injection or lactated Ringer's injection. Vinorelbine was administered prior to topotecan as a slow IV push or rapid IV drip over 8–10 minutes. Topotecan hydrochloride was available as 4 mg vials mixed with mannitol and tartaric acid as excipients. It was then diluted to either 0.025 mg/ml or 0.05 mg/ml in 0.9% sodium chloride or 5% glucose. Topotecan was administered over 30 minutes as an IV infusion subsequent to the infusion of vinorelbine. Agents were administered into a freely running intravenous line.

**Table 1:** Dose Escalation Schema.

	Topotecan (mg/m^2^)	Vinorelbine (mg/m^2^)
Level 1	2	20
Level 2	2.5	20
Level 3	3	20
Level 4	3.5	20
Level 5	4.0	20

### Assessment of response and toxicity

Toxicity was assessed using the NCI Common Toxicity Criteria (version 2.0). If no patients during cycle 1 experienced a dose limiting toxicity (DLT) at a given level, escalation proceeded to the next dose level. A DLT was defined as any grade 3–4 non-hematologic toxicity, grade 4 neutropenia or thrombocytopenia. If one of three patients had a DLT, then three additional patients were to be enrolled in that cohort. If two or more patients had a DLT, that dose was to be declared the Maximum Tolerated Dose (MTD), and the next lower dose level was defined as the recommended phase II dose.

Only those DLTs occurring during the first cycle of therapy were used to define MTD. Those DLTs are described follows: Any documented grade 4 granulocytopenia or thrombocytopenia; any documented ≥ grade 3 non-hematologic toxicity (except grade 3 nausea or vomiting or diarrhea in the first cycle but controlled to maximum of grade 2 with anti-emetic or anti-diarrheal therapy in the second cycle); inability to deliver any chemotherapy on day 8 of cycle 1 because of ANC < 1000/ul or platelet count < 50,000/ul; inability to begin the second cycle of chemotherapy by day 35 because of persisting hematologic or non-hematologic toxicity of ≥ grade 2 and possibly, probably, or clearly due to therapy. Rates of cumulative toxicity were monitored.

Patients received full supportive care measures. The reason(s) for treatment delays and dosage adjustments, and the dates of administration were recorded on flow sheets. Treatment with hormones or other chemotherapeutic agents was prohibited, unless steroids were given for adrenal failure or as intermittent use as anti-emetics. Palliative radiation could not be administered during the treatment protocol.

### Statistical Methods

Time to event outcomes (progression-free survival and overall survival) were analyzed using Kaplan-Meier curves. Median survival estimates were derived and 95% confidence intervals for median survival were calculated using the Greenwood formula.

### Duration of Therapy

Patients with stable or responding disease could continue on therapy at the discretion of the treating physician. For patients who had stable disease after one cycle, but during that cycle experienced a DLT, subsequent protocol therapy could be given at one dose level lower than their assigned starting dose, and further dose reductions could be made. Treatment could continue until disease progression, intercurrent illness that prevented further administration of chemotherapy, unacceptable adverse events, patient decision to withdraw from study, or if general or specific changes occurred in the patient rendering them unacceptable for further treatment in the judgment of the investigator.

### Treatment Assessment

Baseline evaluations were performed within four weeks of beginning therapy using either CT or PET/CT scans. Laboratory evaluation was performed weekly during cycle one, and then on days 1 and 8 of all subsequent cycles. Response and progression was determined by using the Response Evaluation Criteria in Solid Tumors (RECIST). All measurements were recorded in metric notation; imaging to assess response to therapy was obtained every two cycles.

## Results

Eighteen patients with recurrent NSCLC or SCLC were included in this phase I study between February 2004 and March 2007. Patient characteristics are listed in Table 2.

**Table 2:** Patient Characteristics.

**Characteristics**	**n**
Age (years)	
Median	64
Range	49–77
Gender	
Male	9
Female	9
ECOG performance status	
0	5
1	12
2	1
Not Reported	3
Tumor Type	
NSCLC	15
SCLC	3
Previous Chemotherapy Regimens	
1	5
2	2
3	5
≥4	6
Prior Radiotherapy	6

The majority of patients were either asymptomatic or had only mild symptoms. There were nine female and nine male patients. Most of the patients had received at least two primary chemotherapy regimens; only six patients had previously received radiation therapy. All patients had received prior platinum-based chemotherapy, and all but one patient with NSCLC had previously received taxanes. Overall patients enrolled in this study were heavily pretreated with 11/18 patients having received at least 3 prior lines of chemotherapy. The most common tumor type was NSCLC (n = 15), with three patients having recurrent SCLC. The total number of assessable cycles was 75, with the median number of cycles per cohort listed in Table 3.

**Table 3:** Hematologic toxicities. Observed toxicities with vinorelbine/topotecan during all cycles of chemotherapy.

Cohort	N =	Treatment cycles (median)	Neutropenia				Thrombocytopenia		Anemia	
			
			Grade 1–2	Grade 3	Grade 4	FN	Grade 1–2	Grade 3–4	Grade 1–2	Grade 3–4
1	3	12 (4)	1	0	0	0	0	0	7	0
2	3	11 (4)	9	0	0	0	1	0	3	0
3	3	18 (6)	9	6	2*	1*	4	0	10	0
4	6	22 (4)	7	8	1	0	7	0	14	0
5	3	12 (4)	2	0	0	0	2	0	3	0

* Occurred after cycle 1. Abbreviations: FN: febrile neutropenia.

### Hematologic Toxicities

Neutropenia was the most commonly observed grade 3–4 hematologic toxicity, with grade 4 neutropenia occurring in 3 out of 75 total chemotherapy cycles administered (4%). Anemia was frequent, with all events being ≤ grade 2. Thrombocytopenia was also observed, however all events were ≤ grade 2. Febrile neutropenia was reported in one patient in cohort 3 during cycle 4, resulting in hospital admission and treatment with intravenous antibiotics. The MTD was not reached in any of the 5 cohorts. The hematologic toxicity profile is listed in Table 3.

### Non-hematologic Toxicities

There was no evidence of any severe grade 3–4 non-hematologic toxicities in this study (Table 3). The most frequent non-hematologic adverse toxicities with combination vinorelbine/topotecan were grade 1–2 nausea/vomiting, fatigue, constipation, and neuropathy (Table 4). One patient in cohort 2 was taken off of study due to grade 3 fatigue following one cycle of therapy. Transient elevations in hepatic transaminases were also observed in different cohorts. Additional clinically relevant toxicities included febrile illness without neutropenia in three patients, and hyperglycemia in nine patients. Due to the overall tolerability of vinorelbine and topotecan, there were few dose delays with subsequent chemotherapy cycles.

**Table 4:** Non-hematologic toxicities. All toxicities were grade 1–2 unless otherwise specified.

Cohort	N =	Treatment cycles (median)	Fatigue	Nausea/vomiting	Constipation	Alk Phos	Cough/Dyspnea	Hypo-kalemia	Hyper-glycemia	↑ LFTs	Diarrhea	Neuropathy
1	3	12 (4)	2	1	2	1	0	1	2	0	1	1
2	3	11 (4)	2^§^	4	1	0	0	0	3	1	1	2
3	3	18 (6)	6	8	2	3	4**	1	4	2	0	1
4	6	22 (4)	6	2	7	1	1	1	2	1	0	0
5	3	12 (4)	1	3	1	0	0	0	0	0	0	4

§One patient with grade 4 fatigue. **One patient with grade 4 respiratory failure requiring intubation during cycle 2 only.

### Anti-tumor Activity of vinorelbine/topotecan

**Figure 1:**
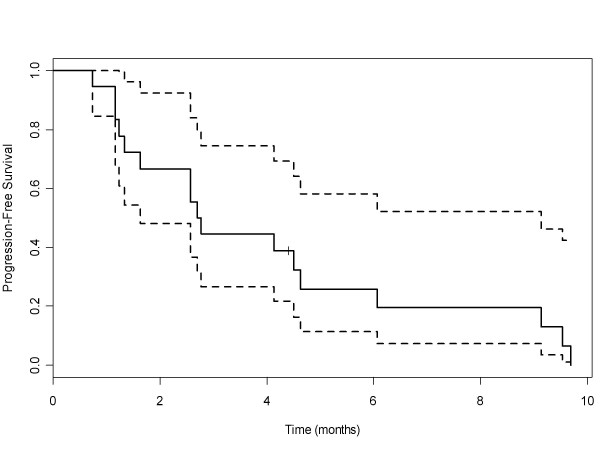
**Progression-Free Survival**. Median progression free survival for patients in all cohorts was 2.7 months (95% CI, 1.6 to 9.1).

**Figure 2:**
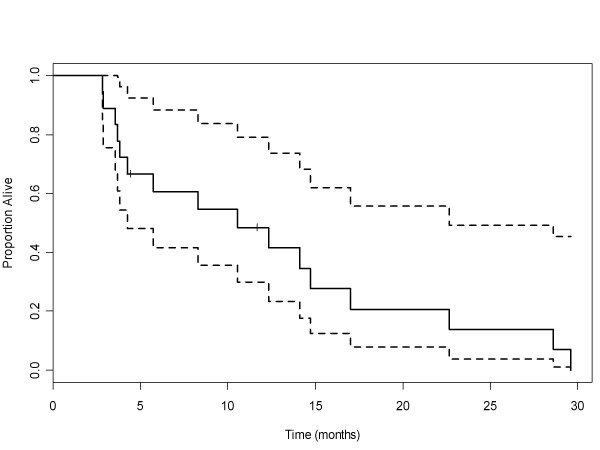
**Overall Survival**. Median overall survival for patients in all cohorts was 10.5 months (95% CI, 4.2 to 22.7)

Fifteen patients were evaluable for tumor response. Only one objective tumor response was observed (partial response); additionally four patients achieved stable disease for ≥ 6 cycles of therapy (Table 5). All patients with stable disease had NSCLC and were heavily pretreated (mean number of previous therapies = 3). Of these patients, one was in cohort 2, two were in cohort 3 and one was in cohort 4. The median follow-up time was 9.4 months. The median progression-free survival and overall survival for all patients were 2.7 months (95% CI: 1.6, 9.1) and 10.5 months (95% CI: 4.2, 22.7), respectively. Figures 1 and 2 demonstrate Kaplan-Meier curves for both progression-free and overall survival, respectively.

**Table 5:** Best Response Rates by cohort.

Cohort	Progressive Disease	Stable Disease	Partial Response	Complete Response
1	3	0	0	0
2	2	1	0	0
3	1	2	0	0
4	5	1	0	0
5	2	0	1	0

## Discussion

Doublet chemotherapy is considered a standard of care for good performance status patients with stage IV NSCLC and extensive stage SCLC; however options for doublet combinations are still limited [4,5,7]. Additional non-platinum and non-taxane doublets are therefore needed. Vinorelbine is well established as an active drug in non-small cell lung cancer, and topotecan is the standard of care for second line therapy for small cell lung cancer. The use of vinorelbine in combination therapy has demonstrated efficacy, particularly in combination with cisplatin. Weekly administration of topotecan has improved tolerability over daily administration [2]. Tolerability of both vinorelbine and topotecan has been well established in advanced lung cancer as single agents [3,13,14]. The different toxicity profiles of vinorelbine and topotecan supports the use of these agents as a non-platinum doublet. Two previous phase I studies have explored the MTD of vinorelbine and topotecan utilizing different schedules than our study. Stupp and colleagues, treated newly diagnosed patients with recurrent or metastatic NSCLC as first-line therapy with topotecan (0.5–1.0 mg/m^2 ^days 1–5) and vinorelbine (20–30 mg/m^2 ^days 1 and 5) in 21-day cycles. Dose-limiting toxicity (DLT) was defined separately with or without the addition of filgrastim. Neutropenia was frequent but transient. Without G-CSF support, the MTD of topotecan was 0.85  mg/m2, with neutropenic fever being the DLT. With the addition of G-CSF,  the MTD was not reached. Non-hematologic toxicities included mild to moderate fatigue and constipation. An overall clinical response rate of 42% was achieved, with responses noted at all dose levels. At a short median follow-up of 15 months, the median survival for all patients was 13 months [29]. In a subsequent phase I trial by Hanna and colleagues, patients with advanced solid tumors with no more than 1 prior chemotherapy regimen were treated with topotecan (1.5 mg/m^2 ^days 1–3) followed by vinorelbine (25 mg/m^2 ^days 1 and 8) without filgrastim every 3 weeks [28]. At this first dose level all three patients experienced hematologic DLT including grade 4 neutropenia grade 3 thrombocytopenia. An additional 2 patients were treated at this same dose level but received prophylactic filgrastim, however both also had DLT. Consequently, alternative schedules to consecutive daily administration of topotecan should be explored both as a single agent and in combination with other agents [16]. To our knowledge, no prior phase I study has been published that investigated the MTD of weekly vinorelbine and topotecan (days 1 and 8) in patients with advanced cancer.

Results from our trial suggest that vinorelbine/topotecan on days 1 and 8 every 21 days is well tolerated. The primary DLT is neutropenia with no grade 3–4 non-hematologic toxicities.

## Conclusion

As the MTD was not achieved in the 5 different dose levels of topotecan in our study, we therefore conclude that the recommended phase II dose for vinorelbine and topotecan, on this particular schedule, is 20 mg/m^2^and 4 mg/m^2^, respectively. Future studies of vinorelbine and topotecan in advanced SCLC and NSCLC are warranted and may provide a better-tolerated alternative to platinum based doublets.

## Competing interests

The author(s) declare that they have no competing interests.

## Authors' contributions

MAB, MRG, MLM: participated in design of clinical trial and writing of clinical protocol; CAS and ASK: enrolled and treated most patients in the study; EGM: was involved in data analysis and creation of survival curves and tables; UC: provided critical review of the manuscript; AJM and MAB: were involved in reviewing clinical data and writing of the manuscript. All authors read and approved the final manuscript.

## Pre-publication history

The pre-publication history for this paper can be accessed here:


